# A retrospective audit of adult and paediatric anaphylaxis management from two Australian metropolitan mixed emergency departments

**DOI:** 10.1186/s12873-024-00966-3

**Published:** 2024-04-17

**Authors:** A Thomas, J Delic, P Hudson, M Batchelor, H Johannsen, LE Grzeskowiak

**Affiliations:** 1 SA Pharmacy, Flinders Medical Centre, Southern Adelaide Local Health Network, Bedford Park, Australia Flinders Drive, 5042; 2 Clinical and Health Sciences, University of South Australia, Adelaide, Australia; 3 Emergency Department, Flinders Medical Centre, Southern Adelaide Local Health Network, Bedford Park, Australia; 4 College of Medicine and Public Health, Flinders University, Adelaide, Australia; 5 Allergy/Clinical Immunology Department, Flinders Medical Centre, Southern Adelaide Local Health Network, Bedford Park, Australia; 6 SAHMRI Women and Kids, South Australian Health and Medical Research Institute, Adelaide, Australia

**Keywords:** Anaphylaxis, Allergy, Emergency department, Immunology

## Abstract

**Background:**

Anaphylaxis is a potentially life-threatening allergic reaction, with presentations to emergency departments (EDs) increasing across Australia. Understanding the features of those presenting with anaphylaxis and aspects related to its optimal clinical management across the admission, treatment and discharge settings is needed to minimise its impact. We aimed to evaluate the nature and management of presentations related to anaphylaxis across two Australian EDs.

**Methods:**

Retrospective audit of paediatric and adult patients presenting to a community or tertiary level ED with anaphylaxis from 1 May 2018 to 30 April 2019. Data extracted from medical records included demographic characteristics, causative agents, clinical features, treatments administered across community, ambulance or ED settings, as well as post-discharge care arrangements including provision of Adrenaline Auto-Injector (AAI) and Allergy/Anaphylaxis Action Plan (AAP).

**Results:**

A total of 369 (107 paediatric and 262 adult) ED presentations were identified. A total of 94 (36%) adult and 46 (43%) paediatric patients received pre-hospital adrenaline, with a further 91 (35%) adult and 29 (27%) paediatric patients receiving a dose of adrenaline in the ED. The most commonly administered treatment in ED were corticosteroids, given to 157 (60%) adult and 55 (51%) paediatric patients. Among those requiring an AAI for discharge, 123/210 (59%) adult and 57/91 (63%) of paediatric patients left hospital with an AAI. In contrast, among those requiring an allergy/anaphylaxis action plan (AAP) on discharge, 61/206 (30%) adult and 30/90 (33%) of paediatric patients left hospital with one. Factors associated with an increased likelihood of receiving AAI on discharge in paediatric and adult patients included receipt of any adrenaline, receipt of two or more doses of adrenaline, and longer duration of hospital stay. Adults presenting within business hours were more likely to be discharged with AAI, but no such difference was observed for paediatric patients. Similar findings were evident for provision of AAP on discharge.

**Conclusion:**

These findings demonstrate the need to improve assessment and treatment in the ED. In particular, the observed large variability in provision of AAI and AAP on discharge presents opportunities to explore strategies to improve awareness and provision of these critical components of post-discharge care.

**Supplementary Information:**

The online version contains supplementary material available at 10.1186/s12873-024-00966-3.

## Background

Anaphylaxis is a severe, potentially life-threatening allergic reaction that requires prompt recognition and immediate treatment to prevent adverse outcomes [1]. In Australia, anaphylaxis presentations to public emergency departments (ED) have grown by 51%, to more than 11,594 over the five year period between 2012–16 to 2019–20, with more than 10,000 presentations occurring each year [2].

With no universally accepted definition, recognising anaphylaxis can be complicated with a reliance on assessing clinical symptoms, with a broad spectrum of presentations possible, even in patients with a history of anaphylaxis [1]. Urgent and effective treatment of anaphylaxis is essential, namely with prompt intramuscular (IM) injection of adrenaline [3]. After treatment commences, care in the ED continues with the focus transitioning to ongoing management of anaphylaxis risk following discharge from hospital. This encompasses educating patients on how to prevent, recognise and respond to future allergic reactions, including the provision of an adrenaline auto-injector (AAI) and allergy/anaphylaxis action plan (AAP), as well as referral to an immunologist [2]. Concerns regarding inadequate recognition and treatment of anaphylaxis acutely in ED, as well as discharge care have led to the recent development of an Australian Acute Anaphylaxis Clinical Care Standard, released in 2021 [2, 4].

The aim of this study was to investigate the assessment and management of patients presenting with anaphylaxis to two mixed EDs.

## Methods

This study involved patients presenting to either Flinders Medical Centre (FMC) ED, a tertiary ED, or the Noarlunga Hospital (NH) ED, a community ED, both located in the Southern Adelaide Local Health Network (SALHN) in Adelaide, Australia. Each department sees approximately 90,000 [5] and 43,0000 adult and paediatric attendances annually respectively [6]. A SALHN guideline for Anaphylaxis Management, providing details on acute and post-discharge care, has been in use in both EDs since 2009 [7]. Numerous multidisciplinary (medical, nursing, pharmacy) campaigns educating ED staff on anaphylaxis management have also run since 2016.

Presentations in the 12-month period between 1 May 2018 and 30 April 2019 for inclusion in the study were identified using International Statistical Classification of Diseases and Related Health Problem codes (ICD-10) discharge codes for anaphylaxis and allergic reactions (Supplemental Table 1). Anaphylaxis specific codes included in our search were T78.0 anaphylaxis and anaphylactic shock due to food, T78.2 anaphylaxis and anaphylactic shock, unspecified, T80.5 anaphylaxis and anaphylactic shock due to serum T88.6 anaphylaxis and anaphylactic shock due to adverse effect of correct drug or medicament properly administered. We also included the following codes to capture potential cases of anaphylaxis miscoded as alternative diagnoses: T78.1 other adverse food reaction, not elsewhere classified, T78.3 angioneurotic oedema, T78.4 other and unspecified allergy, T63.4 toxic effect of venom of other arthropods. Where, available, discharge summaries were reviewed to check the presentation met the eligibility criteria. In situations where the discharge summary was unclear, or where no discharge summary was available, the full medical record was reviewed to assess eligibility.

**Table 1:** Characteristics of individuals presenting to emergency department for anaphylaxis management

	**Paediatric**	**Adult**	**Total**
	(*N* = 107)	(*N* = 262)	(*N* = 369)
**Sex**			
Male	54 (50%)	107 (41%)	161 (44%)
Female	53 (50%)	155 (59%)	208 (56%)
**Median Age, years (range)**	10 (0.5–17)	40 (18–88)	30 (0.5–88)
**Number of ED visits for anaphylaxis during study period**			
1	98 (95%)	232 (94%)	330 (94%)
2	3 (4%)	9 (5%)	12 (5%)
3	1 (1%)	4 (2%)	5 (1%)
**Arrival mode to ED**			
Self	52 (49%)	145 (55%)	197 (53%)
Ambulance	55 (51%)	117 (45%)	172 (47%)
**Initial ED presentation site**			
Tertiary ED	88 (82%)	188 (72%)	276 (75%)
Community ED	19 (18%)	74 (28%)	93 (25%)
**Received adrenaline prior to ED presentation**	46 (43%)	94 (36%)	140 (38%)
**Met ASCIA anaphylaxis criteria prior to arrival to ED**	94 (88%)	240 (92%)	334 (91%)
**Met ASCIA anaphylaxis criteria on arrival to ED**	52 (49%)	159 (61%)	211 (57%)
**Anaphylaxis severity**			
Mild	6 (6%)	10 (4%)	16 (4%)
Moderate	91 (85%)	193 (74%)	284 (77%)
Severe	10 (9%)	59 (22%)	69 (19%)
**Suspected Trigger**			
Food	74 (69%)	95 (36%)	169 (46%)
Venom	7 (7%)	42 (16%)	49 (13%)
Medication	5 (5%)	43 (16%)	48 (13%)
Other	5 (5%)	16 (6%)	21 (6%)
Unknown	16 (15%)	66 (25%)	82 (22%)
**Reacted to previously identified trigger**	51 (53%)	82 (40%)	133 (44%)
**Previous anaphylaxis**	65 (61%)	128 (49%)	193 (52%)
**Previously seen immunologist**	47 (44%)	75 (29%)	122 (33%)
**Comorbid conditions**			
Asthma	34 (32%)	73 (28%)	107 (29%)
Eczema	19 (18%)	12 (5%)	31 (8%)
Allergic rhinitis	3 (3%)	14 (5%)	17 (5%)
Cardiovascular disease	1 (1%)	57 (22%)	58 (16%)
Cognitive impairment	1 (1%)	4 (2%)	5 (1%)
Substance abuse	0 (0%)	11 (4%)	11 (3%)

Patients were included in the study if they (a) presented to ED for treatment of an allergic reaction, and either (b) met Australasian Society of Clinical Immunology and Allergy (ASCIA) criteria for anaphylaxis, or (c) were classified as having anaphylaxis by a treating doctor, or (d) had administered their prescribed adrenaline auto-injector in the pre-hospital setting. The ASCIA definition used in this study is any acute onset illness with typical skin features (urticarial rash or erythema/flushing and/or angioedema), plus involvement of respiratory and/or cardiovascular and/or persistent severe gastrointestinal symptoms; or any acute onset of hypotension or bronchospasm or upper airway obstruction where anaphylaxis is considered possible, even if typical skin features are not present [3]. Patients were excluded if their allergic reaction did not meet the ASCIA definition for anaphylaxis and for which adrenaline was not administered, if the onset of anaphylaxis symptoms did not occur in the community or pre-hospital setting, if an alternative or non-allergy diagnosis was identified, or if there was a known alternative diagnosis (eg hereditary angioedema, idiopathic urticaria).

For each patient, all available data was reviewed including ambulance records, nursing records, medical admission notes, observation charts, hospital pharmacy dispensing records and the hospital referral database. Extracted data included:

- patient demographics, including age, gender, patient/parent medical history, specifically allergic comorbidities (asthma, eczema, allergic rhinitis) and relevant non-allergic comorbidities (cardiovascular disease, substance abuse and cognitive impairment), previous anaphylaxis episodes and if previously seen an immunologist.
- details of the presenting event, including suspected trigger of reaction, mode of transport to hospital, time of presentation, length of hospital stay and discharge destination. Anaphylaxis symptoms, as well as clinical treatments (e.g. adrenaline, antihistamines, corticosteroids, and intravenous fluids) were extracted separately according to pre-hospital (i.e. community, ambulance) and hospital (i.e. ED) settings. The severity of anaphylaxis was classified according to Brown’s grading system for generalised hypersensitivity reactions based on worst symptoms experienced throughout the event [8]. More detailed information was collected during the ED admission with respect to route and frequency of adrenaline doses, and laboratory investigations (i.e. tryptase).
- discharge management including whether or not individuals received a referral to hospital immunologist or a discharge summary was completed, and depending on where relevant, whether allergy status was updated in the medical record, an AAI (or prescription) was provided, and whether an AAP was provided. Where patients were admitted to an inpatient team, the inpatient discharge plans regarding AAP/AAI and immunology referral were recorded instead. Patients were assessed as requiring an AAI or AAP on discharge if they had experienced anaphylaxis according to the ASCIA anaphylaxis definition to an unavoidable/non-medication trigger, and where no current AAP was already in place or where there was no pre-existing supply of AAI.

Descriptive statistics were used to report the characteristics of patients presenting for anaphylaxis and their associated clinical management, stratified by whether they were paediatric (0–17 years) or adult (≥ 18 years). Differences in the likelihood of receiving AAI or AAP according to patient characteristics were compared using Fisher’s Exact Test. A *p*-value < 0.05 was used to determine statistical significance.

Study data were collected and managed using Research Electronic Data Capture (REDCap) hosted at The University of Adelaide [9, 10]. Data were cleaned and analysed using STATA 16 (StataCorp LP, College Station, TX). Figures were prepared using GraphPad Prism 9 or R, version 4.3.0 (R Core Team). Ethics approval was obtained from the Southern Adelaide Clinical Human Research Ethics Committee (OFR: 120.19). The need for individual participant consent was waived by the ethics committee as this was a deemed a low-risk retrospective audit of medical records.

## Results

### Study cohort

There were 926 patient presentations to either emergency department (ED) identified using ICD-10 codes discharge codes for anaphylaxis and allergic reactions. Around half (*n* = 538) were excluded for not meeting the ASCIA definition of anaphylaxis. Additionally, 10 presentations were identified as duplicate ED encounters for the same anaphylaxis episode, with another 9 paper medical records unable to be reviewed. This left a total of 369 ED presentations (*n* = 347 unique patients) meeting the inclusion criteria.

### Cohort characteristics

Demographic characteristics of the study cohort are presented in Table 1. Two-thirds of presentations were adults (262; 71%). The median age for adult patients was 40 years (Range 18–88 years) and 10 years (Range 6 months to 17 years) for paediatric patients. Approximately half of adult (117; 45%) and paediatric patients (55; 51%) arrived by ambulance. Most paediatric patients (65, 61%) had a history of anaphylaxis, with the majority of these known to an immunologist (47; 44%). Approximately a third of adult (73; 28%) and paediatric patients (34; 32%) also had asthma. The second most common co-morbidity in paediatric patients was eczema (18; 19%), compared with cardiovascular disease in adults (57; 22%). Food was the most commonly suspected trigger among paediatric (74, 69%) and adult patients (95, 36%). Approximately half of paediatric (51, 53%) and adult patients (82, 40%) reacted to a previously known trigger.

### Clinical features of anaphylaxis

Clinical features of anaphylaxis in the study cohort are presented in Table 2. Skin and respiratory symptoms were the most common clinical features, experienced by 235 (90%) and 225 (86%) of adults respectively, and 91 (85%) and 96 (90%) of paediatric patients respectively. Cardiovascular symptoms were evident in 153 (58%) adults, compared with just 38 (36%) paediatric patients. The combination of affected body systems during episodes of anaphylaxis are presented in Supplemental Fig. 1 and Supplemental Fig. 2. The most frequently affected combination of body systems in paediatric patients were respiratory and skin symptoms (29; 27%), followed by skin, respiratory and gastrointestinal symptoms (23; 21%). Adult patients most commonly reported symptoms in all four systems (64; 24%), followed by skin and respiratory symptoms (56; 22%).

**Table 2:** Clinical features in individuals presenting to emergency department for management of anaphylaxis

Symptoms, n (%)	Paediatric	Adult	Total
	(*N* = 107)	(*N* = 262)	(*N* = 369)
**Skin**			
Any	91 (85%)	235 (90%)	326 (88%)
Urticaria	60 (56%)	144 (55%)	204 (55%)
Pruritis	56 (52%)	144 (55%)	200 (54%)
Angioedema/swollen lips	53 (50%)	142 (54%)	195 (53%)
Generalised erythema	38 (36%)	101 (39%)	139 (38%)
Swollen tongue	20 (19%)	61 (23%)	81 (22%)
Periorbital swelling	26 (24%)	50 (19%)	76 (21%)
**Respiratory**			
Any	96 (90%)	225 (86%)	321 (87%)
Swelling/tightness in throat	52 (49%)	127 (48%)	179 (49%)
Shortness of breath	41 (38%)	123 (47%)	164 (44%)
Chest tightness	21 (20%)	73 (28%)	94 (25%)
Wheeze	26 (24%)	67 (26%)	93 (25%)
Difficulty talking/hoarseness	25 (23%)	56 (21%)	81 (22%)
Difficult/noisy breathing	25 (23%)	47 (18%)	72 (20%)
Cough	23 (21%)	22 (8%)	45 (12%)
Hypoxia (≤ 92%)	7 (7%)	26 (10%)	33 (9%)
Stridor	8 (7%)	9 (3%)	17 (5%)
**Cardiovascular**			
Any	38 (36%)	153 (58%)	191 (52%)
Dizziness/light-headedness	11 (10%)	92 (35%)	103 (28%)
Tachycardia	20 (19%)	62 (24%)	82 (22%)
Hypotension	2 (2%)	47 (18%)	49 (13%)
Diaphoresis	7 (7%)	33 (13%)	40 (11%)
Pallor	4 (4%)	14 (5%)	18 (5%)
Confusion	5 (5%)	12 (5%)	17 (5%)
Loss of consciousness	1 (1%)	12 (5%)	13 (4%)
Mottled periphery	3 (3%)	2 (1%)	5 (1%)
**Gastrointestinal**			
Any	50 (47%)	126 (48%)	176 (48%)
Nausea/vomiting	37 (35%)	114 (44%)	151 (41%)
Abdominal cramps	24 (22%)	44 (17%)	68 (18%)
Urgency for the toilet/diarrhoea	2 (2%)	25 (10%)	27 (7%)

Totals do not sum to 100% as people could report multiple symptoms

**Fig. 1:**
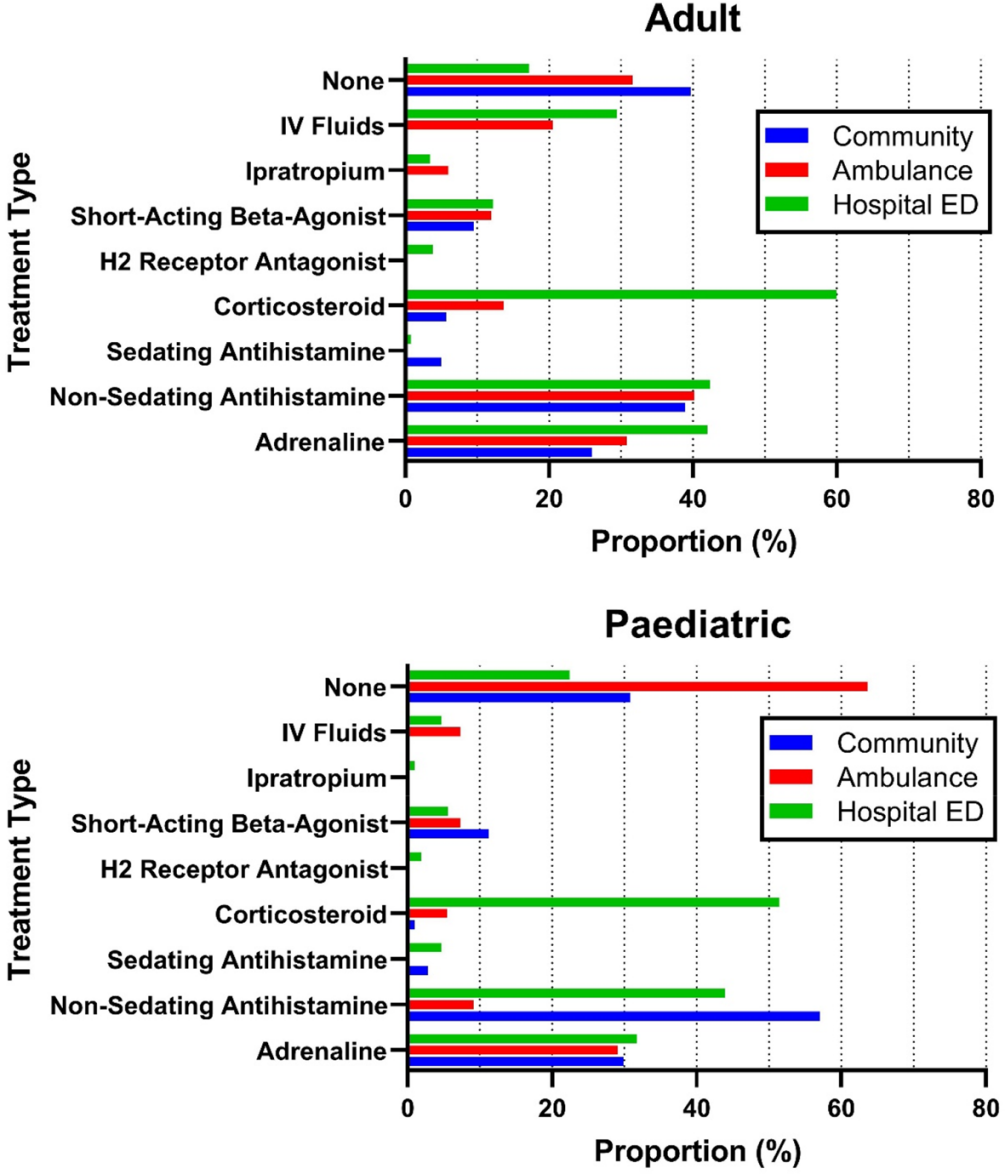
Treatment administered to patients presenting to emergency department for management of anaphylaxis

### Clinical management

The proportion of patients receiving different treatments for anaphylaxis is presented in Fig. 1, stratified according to treatment setting. Non-sedating antihistamines were the most administered treatment in the community setting (102; 39%) and in adults transported to hospital by ambulance (47; 40%). The majority of paediatric patients did not receive any medications administered by the ambulance service (34; 64%;). The proportion of paediatric patients receiving adrenaline was similar across each treatment setting (29% to 32%). In contrast, a large difference in adrenaline use in adult patients was evident across treatment settings, ranging from 68 (26%) in the community to 110 (42%) in ED. Overall, 185 (71%) of adult patients and 75 (70%) paediatric patients received treatment with adrenaline across the pre-hospital and ED setting, with 19 (7%) adult patients and 5 (5%) paediatric patients receiving adrenaline across both the pre-hospital and ED settings. Corticosteroids were the most common medications administered in ED, given to 157 (60%) adult and 55 (51%) paediatric patients. Nearly a third (77; 29%) of adult patients received IV fluid, compared to 5 (5%) paediatric patients. A total of 52 (20%) adult and 24 (22%) paediatric patients did not receive any medication in ED at all. Notably, while 94 (88%) of paediatric patients and 240 (92%) of adult patients met the ASCIA criteria for anaphylaxis in the pre-hospital setting, only 46 (43%) of paediatric patients and 94 (36%) of adult patients received treatment with adrenaline prior to arrival to the ED. Among patients who met the ASCIA criteria for anaphylaxis on arrival to ED, 91/159 (57%) of adult and 29/52 (56%) of paediatric patients received adrenaline in ED. The median time to adrenaline given by any route in symptomatic adult patients with no pre-hospital adrenaline was 14 min (1–94 min), with 50/52 (96%) receiving doses within 1 h of ED triage. The median time in paediatric patients was higher at 20 min (6–98 min), with 15/16 (94%) receiving doses within 1 h of ED triage.

Details on ED management are presented in Table 3. Adrenaline was most commonly administered via the IM route in ED, however a small number of patients received adrenaline via nebuliser, intravenous (IV) infusion or multiple routes. Notably, 48 (45%) paediatric and 78 (30%) of adult patients were discharged from ED within 4 hours of arrival. The median length of stay for paediatric patients was shorter than adults, at 4.4 hours (IQR 2.8–14.2 h) compared to 5.8 hours (IQR 3.8–11.2 h). Twenty-one (8%) adults and 36 (34%) paediatric patients were admitted to an inpatient unit. Blood pathology was ordered in more adult (180; 69%) than paediatric patients (24; 22%), however a mast cell tryptase was ordered in only 113 (43%) of adult and 19 (18%) paediatric patients. Patients were less likely to have a tryptase ordered if they had a history of anaphylaxis (75% vs 52%, *P* < 0.001) or had reacted to a previously known trigger (79% vs 59%, *P* < 0.001). An allergy clinic referral was completed for one third (35; 33% of paediatrics, 99; 38% of adults) of patients and a discharge summary was available for 70 (65%) of paediatric and 99 (38%) of adult presentations.

**Table 3:** Management of individuals presenting to emergency department with anaphylaxis

	**Paediatric**	**Adult**	**Total**
	(*N* = 107)	(*N* = 262)	(*N* = 369)
**Treatment with adrenaline in ED**	34 (32%)	110 (42%)	144 (39%)
**Received ≥ 2 of adrenaline in ED**	8 (7%)	19 (7%)	27 (8%)
**Route of adrenaline administration**			
IM only	26 (76%)	91 (83%)	117 (82%)
Nebulised only	6 (18%)	6 (5%)	12 (8%)
IV infusion only	0	1 (1%)	1 (1%)
IM + nebulised	2 (6%)	7 (6%)	9 (6%)
IM + IV infusion	0	3 (3%)	3 (2%)
Nebulised + IV infusion	0	1 (1%)	1 (1%)
IM + IV infusion + nebulised	0	1 (1%)	1 (1%)
**Median time to adrenaline**^**a**^**, mins (min–max)**	20 (6–98)	14 (1–94)	15 (1–94)
**Required transfer to tertiary ED**	3 (3%)	10 (4%)	13 (4%)
**Disposition from ED**			
Discharge from ED	71 (66%)	79 (30%)	150 (40%)
Emergency Short Stay Unit	0	162 (62%)	162 (44%)
Inpatient team	36 (34%)	14 (5%)	50 (14%)
Intensive care unit	0	6 (2%)	6 (2%)
Transfer to private hospital	0	1 (1%)	1 (< 1%)
**Hospital length of stay**			
< 4 h	48 (45%)	78 (30%)	126 (34%)
4–12 h	28 (26%)	121 (46%)	149 (40%)
12–24 h	28 (26%)	41 (16%)	69 (19%)
24 + hours	3 (3%)	21 (8%)	24 (7%)
**Median length of stay (hours)**	4.4 (2.8–14.2)	5.8 (3.8–11.2)	5.3 (3.6–12.0)
**Tryptase ordered**	19 (18%)	113 (43%)	132 (36%)
**Any blood pathology ordered**	24 (22%)	180 (69%)	204 (55%)
**Hospital immunologist referral**	35 (33%)	99 (38%)	134 (36%)
**Discharge summary completed**	70 (65%)	219 (84%)	289 (79%)
**Allergy status updated in medical record**			
Yes	12 (11%)	53 (20%)	65 (18%)
No	74 (69%)	132 (51%)	206 (56%)
Not applicable—previously documented in medical record	5 (5%)	10 (4%)	15 (4%)
Not applicable—trigger unknown	16 (15%)	66 (25%)	82 (22%)
**Given adrenaline autoinjector on discharge**	61 (60%)	137 (52%)	198 (54.4%)
Yes	57 (53%)	123 (47%)	180 (49%)
No	34 (32%)	87 (33%)	121 (33%)
Not indicated/required	16 (15%)	52 (20%)	68 (18%)
**Given allergy/anaphylaxis action plan on discharge**			
Yes	30 (28%)	61 (23%)	91 (25%)
No	60 (56%)	145 (55%)	205 (56%)
Not indicated/required	17 (16%)	56 (22%)	73 (19%)

^a^calculated in those not previously administered adrenaline in the pre-hospital setting

Among those requiring an AAI for discharge, 68% (*n* = 123/210) of adult patients and 63% (*n* = 57/91) of paediatric patients left hospital with an AAI, either supplied from the hospital or as a discharge script. Factors associated with increased likelihood of receiving an AAI on discharge included symptoms consistent with anaphylaxis on arrival to ED, or a deterioration of symptoms in ED, the use of any adrenaline, including pre-hospital or in ED, requiring 2 or more adrenaline doses, discharging from an inpatient unit, length of stay in hospital, and presenting in business hours (all *P* < 0.05) (Table 4). Findings were similar when examined separately for adult and paediatric patients, except there was no difference in AAI provision on discharge according to discharge unit for adult patients, or time of day presenting to the ED or deterioration of symptoms in paediatric patients. Further, paediatric patients reporting pre-hospital symptoms had an increased likelihood of being discharged with an AAI (Supplemental Table 2).

**Table 4:** Factors Associated with supply of Adrenaline Autoinjector (AAI) and Allergy/Anaphylaxis Plan (AAP) on hospital discharge

	**AAI supplied on discharge**^**a**^		***p*-value**	AAP supplied on discharge^a^		***p*-value**
	**Yes**	**No**		**Yes**	**No**	
	*N* = 180	*N* = 121		*N* = 91	*N* = 205	
**Sex**			0.098			0.313
Male	88 (49%)	47 (39%)		45 (49%)	88 (43%)	
Female	92 (51%)	74 (61%)		46 (51%)	117 (57%)	
**Age category**			0.525			0.584
Paediatric	57 (32%)	34 (28%)		30 (33%)	60 (29%)	
Adult	123 (68%)	87 (72%)		61 (67%)	145 (71%)	
**History of anaphylaxis**	101 (56%)	64 (53%)	0.637	32 (35%)	121 (59%)	< 0.001
**Comorbid conditions**						
Asthma	59 (33%)	35 (29%)	0.527	32 (35%)	58 (28%)	0.274
Eczema	17 (9%)	8 (7%)	0.523	8 (9%)	19 (9%)	1.000
Allergic rhinitis	11 (6%)	4 (3%)	0.419	3 (3%)	12 (6%)	0.566
Cardiovascular disease	30 (17%)	11 (9%)	0.086	17 (19%)	24 (12%)	0.144
**Reacted to previously identified trigger**	75 (50%)	45 (50%)	1.000	29 (38%)	82 (52%)	0.051
Suspected Trigger*			0.167			0.009
Food	99 (55%)	57 (47%)		48 (53%)	103 (50%)	
Venom	31 (17%)	16 (13%)		23 (25%)	25 (12%)	
Unknown	41 (23%)	37 (31%)		17 (19%)	60 (29%)	
Other	9 (5%)	11 (9%)		3 (3%)	17 (8%)	
**Anaphylaxis symptoms**						
Pre**-**hospital	174 (97%)	112 (93%)	0.175	82 (90%)	186 (91%)	0.833
On arrival to ED	114 (63%)	61 (50%)	0.032	67 (74%)	102 (50%)	< 0.001
Deterioration in ED	27 (15%)	7 (6%)	0.015	22 (24%)	13 (6%)	< 0.001
**Received adrenaline**						
Any time	160 (89%)	47 (39%)	< 0.001	83 (91%)	121 (59%)	< 0.001
Pre-hospital	94 (52%)	20 (17%)	< 0.001	34 (37%)	74 (36%)	0.896
ED	80 (44%)	31 (26%)	< 0.001	58 (64%)	54 (26%)	< 0.001
**Total adrenaline doses**			< 0.001			< 0.001
0	20 (11%)	74 (61%)		8 (9%)	84 (41%)	
1	114 (63%)	38 (31%)		55 (60%)	100 (49%)	
≥ 2	46 (26%)	9 (7%)		28 (30%)	21 (9%)	
**Presented in business hours** **(Mon-Fri 8am-4 pm)**	89 (49%)	37 (31%)	0.001	64 (70%)	56 (27%)	< 0.001
**Length of stay in ED/hospital**			< 0.001			< 0.001
< 4 h	49 (27%)	57 (47%)		12 (13%)	93 (45%)	
4–12 h	75 (42%)	49 (40%)		29 (32%)	86 (42%)	
12–24 h	46 (26%)	11 (9%)		44 (48%)	18 (9%)	
24 + hours	10 (6%)	4 (3%)		6 (7%)	8 (4%)	
**Discharge Unit**			0.010			< 0.001
ED	147 (82%)	112 (93%)		64 (70%)	187 (91%)	
Inpatient Unit	33 (18%)	9 (7%)		27 (30%)	18 (9%)	

^a^ Cohort excludes those where provision of AAI or AAP on discharge was not indicated or required

*patients with medication trigger excluded from cohort

Among those requiring an AAP on discharge, 30% (*n* = 61/206) of adult patients and 33% (*n* = 30/90) of paediatric patients received one. Factors associated with an increased provision of AAP were symptoms consistent with anaphylaxis on arrival to ED, deterioration of symptoms in ED, treatment with adrenaline in ED, and presenting in business hours (Table 4). Findings were similar when examined separately for adult and paediatric patients, except there was no difference in AAP provision according to discharge unit or if the patient had reacted to a previously identified trigger in adult patients (Supplemental Table 3).

## Discussion

In this large audit of 107 paediatric and 262 adult patients presenting to ED with anaphylaxis, we found that IM adrenaline remains underutilised for anaphylaxis treatment and provision of AAI, AAP and referral to immunologist on discharge remains suboptimal. Efforts to improve early recognition and management of anaphylaxis in the ED are warranted, as are strategies to improve discharge management.

The identified rate of adrenaline administered to those presenting with anaphylaxis to ED in our study (57%) was within the range of 39% to 59% reported in previous studies [11–14]. Adrenaline is the first-line treatment for anaphylaxis and should be given without delay via the IM route [3]. Previous studies have shown that alternate routes of adrenaline administration are frequently used, with De silva [15] describing 52% of doses given via subcutaneous (SC) injection and Brown [16] reporting 70% patients treated with IV adrenaline in ED. The IM route was predominately used in our study, with no use of the SC route identified in the ED. However, nearly one fifth of paediatric patients (6; 18%) received adrenaline via nebuliser only, all being 10 years or older. While nebulised adrenaline may be used as an adjunct to IM adrenaline in anaphylaxis for upper airway obstruction or bronchospasm, it must not be used in preference to IM adrenaline due to negligible systemic absorption [1, 3, 17]. Our nebulised adrenaline use is higher than the 2.3% of paediatric patients reported to receive inhaled adrenaline by Andrews [18], a study of pre-hospital paediatric anaphylaxis management in Victoria, Australia between 2008–2016. It also contrasts with Brown [16], De Silva [15] and Nogic [12], who did not identify nebulised adrenaline use in their population, despite reporting on alternative adrenaline routes and other inhaled medications administered. The use of nebulised adrenaline in this older paediatric cohort suggests that there may be hesitancy to use IM adrenaline in this age group or confusion regarding the ideal route of administration, rather than anaphylaxis initially being diagnosed as croup, as may happen in younger children. Details of the treating doctor experience level or paediatric-specific qualifications was not collected and therefore we were unable to investigate what impact it may have on this prescribing practice.

Corticosteroids were found to be the most administered treatment in our EDs, with over half of adult and paediatric patients receiving at least one dose. This corticosteroid use is similar to other Australian ED studies, with use reported to range from 44 to 87% [11–16, 19]. The evidence supporting the routine use of corticosteroids in anaphylaxis management remains a subject of debate, with increasing evidence of no benefit in the acute management phase or in preventing biphasic reactions [20]. Additionally, evidence of potential harms has been reported in Gabrielli [21], where pre-hospital use of corticosteroids was associated with increased risk of ICU admission.

AAI were provided to 63% of eligible paediatric and 68% of eligible adult patients on discharge. This is in line with Loprete, who reported 67.9% of their adult patients discharged with an AAI across a 10 year period in their single adult only ED [14]. However, other contemporary Australian studies undertaken in paediatric or mixed EDs have reported much lower rates of AAI provision on discharge ranging from 25 to 35% [11–13]. While subsided AAI prescriptions have been available since 2003 via the Pharmaceutical Benefit Scheme (PBS), an Australian Government program that subsidises the costs of medications for patients meeting eligibility criteria, ED doctors were unable to prescribe AAI for new anaphylaxis patients until 2006 [22, 23]. Currently, the initial supply of a subsidised AAI is restricted to patients who are being discharged from hospital or ED after treatment with adrenaline for anaphylaxis; or patients assessed at being at risk of anaphylaxis in consultation with a clinical immunologist, allergist, paediatrician or respiratory physician [24]. Therefore, it is unsurprising that any adrenaline use is associated with an increased likelihood of receiving AAI on discharge and ineligibility for subsidised AAI is an unintended consequence of not using adrenaline to treat anaphylaxis in ED. Access to additional clinical services, such as hospital immunology consultations and the clinical pharmacy service to ED, during weekdays may explain the association with presenting time and length of stay, with increased likelihood of AAI on discharge in adult patients. This association was not seen in paediatric patients, who had higher rates of admission to an inpatient team and therefore discharged from the care of a paediatrician.

Only two prior studies have evaluated AAP provision on discharge from ED following presentation for anaphylaxis. These observed large variation in rates of AAP provision on discharge of 6% and 41% respectively, with our study observing a rate of 31% [11, 14]. Similar to AAI, presenting in business hours was also associated with increased likelihood of discharge with an AAP. Again, this may relate to the hours the ED clinical pharmacy service operates, who are involved in identifying patients in need of an AAP on discharge when involved in the supply and education of patients receiving a discharge AAI. Further, only 36% of patients received a referral to an immunologist, within the range of 9% to 55% reported in other Australian studies [12–14, 16, 19]. However, the discrepancy between AAI and AAP provision with low immunology referrals, raises the concern that patients may be discharging home with an AAI but lacking clear written information on how to recognise anaphylaxis and initiate appropriate treatment, with no clear follow-up plan to resolve these management issues leaving the patient vulnerable and also heightening the impact on the patient and their families' quality of life. Additionally, patients who are not referred to immunology for follow-up may miss the opportunity for a comprehensive review of their anaphylaxis risk profile and the potential consideration of treatment options, including venom desensitisation.

We found that approximately one-third (34%) were discharged from the ED within 4 hours of arrival, forgoing the suggested minimum 4 hours of observation [2, 3] in case of anaphylaxis recurrence. Very few studies report on length of observation or length of hospital stay. In an early study by Brown, all patients stayed for at least 4 hours, with the median length of stay of patients who were discharged directly from ED at 6 h:32 min (range 4:01 to 9:15) [16]. More recently in 2016, Murad reported patients discharging prior to 4 h at 46% [13]. This was despite an educational intervention aimed at improving anaphylaxis management in their ED, with an increase of 11% of patients discharging early in their post-intervention cohort, with increasing bed demands in the ED felt to be the main contributor to this. The impacts of increasing hospital bed demands that have occurred in the past decade are demonstrated by Loprete who have observed steady increases in the proportion of patients discharged within 4 h from 36% between January 2009-April 2013, to 44% between May 2013-October 2016, and 47% between November 2016-December 2018 [14].

Another aspect of ED care reviewed included the ordering of blood tests, specifically serum tryptase levels. Tryptase is released from mast cell granules during anaphylaxis, with serum levels rising and peaking 1–2 hours after anaphylaxis, returning to normal levels at approximately 6 hours [25]. Therefore, an ED tryptase level may assist with confirming or challenging an anaphylaxis diagnosis when reviewed by an immunologist [1, 17]. Consensus guidelines recommend timed serial tryptase levels are taken after a suspected anaphylactic reaction, as soon as possible after emergency treatment has commenced, with a second level ideally taken within one to two hours (but no later than 4 h) from the onset of symptoms [26]. Alternatively, if this is missed, a single sample taken up to six hours may be taken instead, albeit potentially less helpful [27]. Other blood tests ordered in ED, such as EUC, CBE, LFTs and CRP, have limited clinical utility in anaphylaxis patients [25]. Our study identified that while blood tests were commonly ordered in adult patients, a tryptase level was only measured in 63% of these patients, all by single sample. Conversely, blood tests were ordered in only 22% of paediatric patients, with all but five patients having their serum tryptase measured by single sample. To our knowledge, the rates and types of blood tests ordered in patients presenting with anaphylaxis to ED has not been reported previously in Australia. Our findings, particularly the discrepancy between adult and paediatric blood tests ordered, identifies the potential for a large number of unnecessary blood tests being ordered in adults, as well as a lost opportunity in capturing the acute rise of tryptase for immunologist review.

### Strengths and limitations

To our knowledge, this is the largest contemporary study of both adult and paediatric anaphylaxis management and discharge care, conducted in Australia. Previous studies have predominantly examined care in either adult or paediatric patients and their respective ED settings, with the only studies examining care in this combined cohort restricted to fewer than 100 cases, did not examine provision of AAI or AAP on discharge or were conducted prior 2006, following which major changes were made to enable ED doctors to prescribe subsidised AAI for patients discharging with anaphylaxis [11–16, 19, 22, 28].

This study has a number of limitations. Firstly, it was conducted in a single hospital network in a metropolitan population, limiting generalisability of the findings to other settings. Using ICD codes to identify anaphylaxis presentations introduces potential for misclassification. We attempted to capture any miscoded anaphylaxis cases by including ICD codes assigned to other non-allergic reactions, however did not have the capacity to search through the volume of presentations assigned ICD codes related to urticaria or asthma. The nature of a retrospective case note review meant that data collection relied heavily on clinician documentation, particularly in key areas such as signs and symptoms used to classify the reaction severity, suspected trigger, as well as the post-discharge care arranged. Data was collected from a variety of sources available, including ambulance records, the patient history as recorded by each health practitioner seeing the patient, the triage note, treating doctor’s notes, any nursing notes, discharge note, hospital discharge letters and referral forms completed. Future research utilising prospective and more comprehensive data collection methods may help address some of these limitations and provide a more robust understanding of anaphylaxis management in the ED setting.

## Conclusion

Anaphylaxis presentations to ED continue to have suboptimal use of intramuscular adrenaline and high use of corticosteroids. Post-discharge care, including discharge with an AAI, AAP and referral to immunologist, also demonstrates room for improvement, particularly in patients who present to ED out of hours with reduced other clinical services available.

### Supplementary Information

**Additional file 1. Supplemental Table 1. **ICD-10 codes used to identify potential anaphylaxis presentations to ED.

**Additional file 2. Supplemental Figure 1. **Combinations of clinical features of anaphylaxis by body systems in adult patients presenting with anaphylaxis to emergency department.

**Additional file 3. Supplemental Figure 2. **Combinations of clinical features of anaphylaxis by body systems in paediatric patients presenting with anaphylaxis to emergency department.

**Additional file 4. Supplemental Table 2. **Factors Associated with supply of Adrenaline Autoinjector (AAI) on discharge from hospital in paediatric and adult patients.

**Additional file 5. Supplemental Table 3. **Factors Associated with supply of Allergy/Anaphylaxis Action Plans (AAP) on discharge from hospital in paediatric and adult patients.

## Data Availability

The datasets used and/or analysed during the current study are available from the corresponding author on reasonable request.

## Abbreviations

AAI: Adrenaline autoinjector
AAP: Allergy/Anaphylaxis action plan
ASCIA: Australasian Society of Clinical Immunology and Allergy
CBE: Complete blood examination
CRP: C-reactive protein
ED: Emergency department
EUC: Electrolytes, urea and creatinine
IM: Intramuscular
IV: Intravenous
LFT: Liver function test
SC: Subcutaneous
