# Supplementary material for: A retrospective audit of adult and paediatric anaphylaxis management from two Australian metropolitan mixed emergency departments
Source: BMC Emerg Med. 2024 Apr 17;24:67. doi: 10.1186/s12873-024-00966-3 (PMC11022440; doi:10.1186/s12873-024-00966-3)
Supplement: Supplementary file 1 — Additional file 1. Supplemental Table 1. ICD-10 codes used to identify potential anaphylaxis presentations to ED. [file 12873_2024_966_MOESM1_ESM.docx]

**Supplemental Table 1. ICD-10 codes used to identify potential anaphylaxis presentations to ED**

| T78.0 | Anaphylaxis and anaphylactic shock due to food |
| --- | --- |
| T78.2 | Anaphylaxis and anaphylactic shock, unspecified |
| T80.5 | Anaphylaxis and anaphylactic shock due to serum |
| T88.6 | Anaphylaxis and anaphylactic shock due to adverse effect of correct drug or medicament properly administered |
| T78.1 | Other adverse food reaction, not elsewhere classified |
| T78.3 | Angioneurotic oedema |
| T78.4 | Other and unspecified allergy |
| T63.4 | Toxic effect of venom of other arthropods |
