# Supplementary material for: A retrospective audit of adult and paediatric anaphylaxis management from two Australian metropolitan mixed emergency departments
Source: BMC Emerg Med. 2024 Apr 17;24:67. doi: 10.1186/s12873-024-00966-3 (PMC11022440; doi:10.1186/s12873-024-00966-3)

**Supplemental Figure 1. Combinations of clinical features of anaphylaxis by body systems in adult patients presenting with anaphylaxis to emergency department**


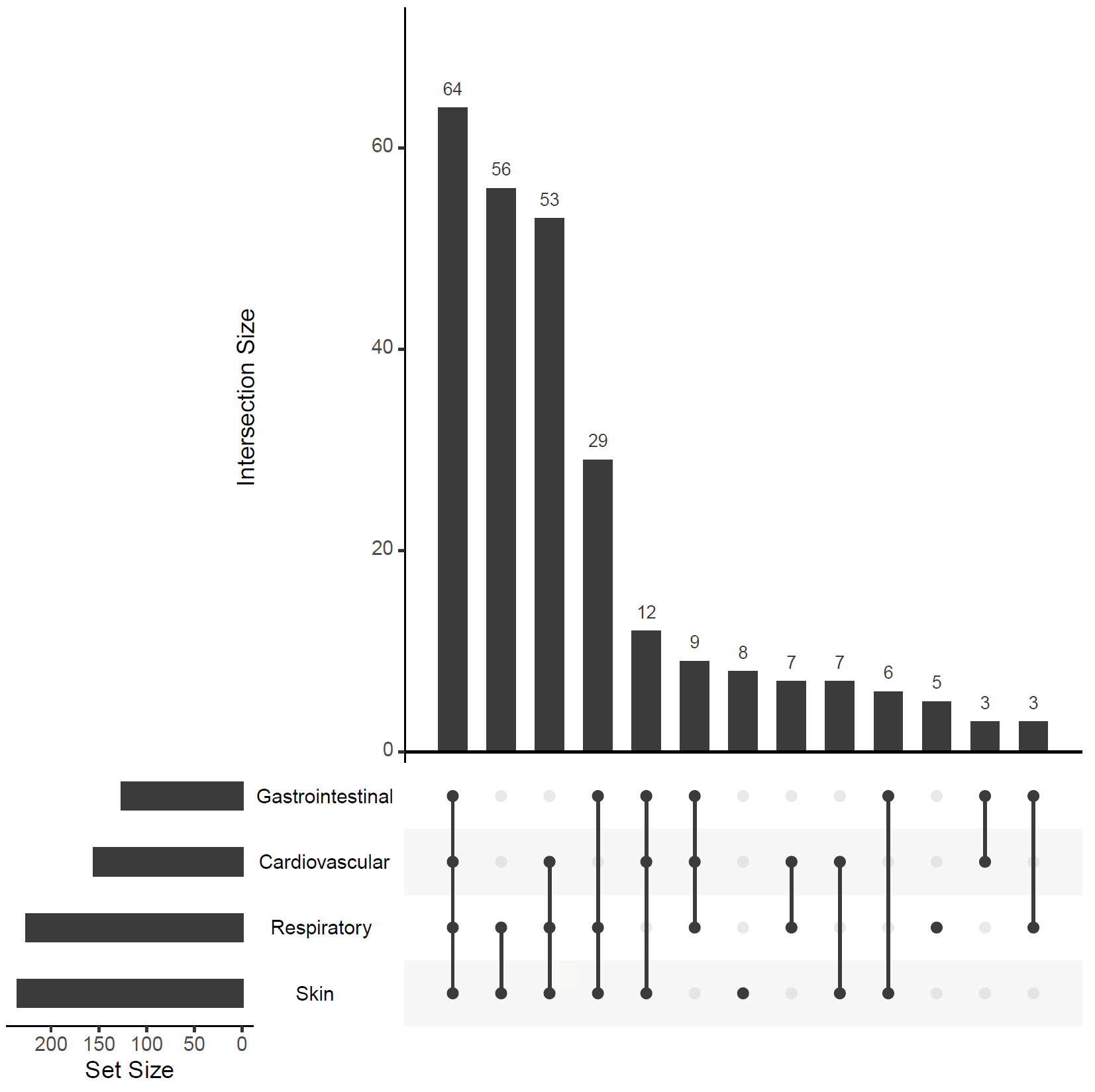

Supplement: Supplementary file 2 — Additional file 2. Supplemental Figure 1. Combinations of clinical features of anaphylaxis by body systems in adult patients presenting with anaphylaxis to emergency department. [file 12873_2024_966_MOESM2_ESM.docx]
