# Supplementary material for: A retrospective audit of adult and paediatric anaphylaxis management from two Australian metropolitan mixed emergency departments
Source: BMC Emerg Med. 2024 Apr 17;24:67. doi: 10.1186/s12873-024-00966-3 (PMC11022440; doi:10.1186/s12873-024-00966-3)
Supplement: Supplementary file 4 — Additional file 4. Supplemental Table 2. Factors Associated with supply of Adrenaline Autoinjector (AAI) on discharge from hospital in paediatric and adult patients. [file 12873_2024_966_MOESM4_ESM.docx]

**Supplemental Table 2. Factors Associated with supply of Adrenaline Autoinjector (AAI) on discharge from hospital in paediatric and adult patients**

| \|  \| **Paediatric** \| \| \| **Adult** \| \| \| \| --- \| --- \| --- \| --- \| --- \| --- \| --- \| \|  \| **AAI supplied on discharge†** \| \| **p-value** \| **AAI supplied on discharge†** \| \| **p-value** \| \|  \| Yes \| No \|  \| Yes \| No \|  \| \|  \| N= 57 \| N=34 \|  \| N=123 \| N=87 \|  \| \| **Sex** \|  \|  \| 0.278 \|  \|  \| 0.259 \| \| Male \| 33 (58%) \| 15 (44%) \|  \| 55 (45%) \| 32 (37%) \|  \| \| Female \| 24 (42%) \| 19 (56%) \|  \| 68 (55%) \| 55 (63%) \|  \| \| **History of Anaphylaxis** \| 38 (67%) \| 21 (62%) \| 0.656 \| 63 (51%) \| 43 (49%) \| 0.889 \| \| **Comorbid conditions** \|  \|  \|  \|  \|  \|  \| \| Asthma \| 19 (33%) \| 11 (32%) \| 1.000 \| 40 (33%) \| 24 (28%) \| 0.543 \| \| Eczema \| 12 (21%) \| 3 (9%) \| 0.155 \| 5 (4%) \| 5 (6%) \| 0.744 \| \| Allergic Rhinitis \| 1 (2%) \| 2 (6%) \| 0.553 \| 10 (8%) \| 2 (2%) \| 0.128 \| \| Cardiovascular disease \| 1 (2%) \| 0 (0%) \| 1.000 \| 29 (24%) \| 11 (13%) \| 0.051 \| \| Cognitive impairment \| 1 (2%) \| 0 (0%) \| 1.000 \| 1 (1%) \| 2 (2%) \| 0.571 \| \| Substance Abuse \|  \|  \| . \| 4 (3%) \| 5 (6%) \| 0.494 \| \| **Reacted to previously identified trigger** \| 30 (56%) \| 18 (67%) \| 0.472 \| 45 (47%) \| 27 (43%) \| 0.626 \| \| **Suspected Trigger** \|  \|  \| 0.085 \|  \|  \| 0.300 \| \| Food \| 44 (77%) \| 21 (62%) \|  \| 55 (45%) \| 36 (41%) \|  \| \| Venom \| 5 (9%) \| 2 (6%) \|  \| 26 (21%) \| 14 (16%) \|  \| \| Unknown \| 5 (9%) \| 10 (29%) \|  \| 36 (29%) \| 27 (31%) \|  \| \| Other \| 3 (5%) \| 1 (3%) \|  \| 6 (5%) \| 10 (11%) \|  \| \| **Anaphylaxis symptoms** \|  \|  \|  \|  \|  \|  \| \| Pre-hospital \| 31 (54%) \| 7 (21%) \| 0.002 \| 120 (98%) \| 81 (93%) \| 0.167 \| \| On arrival to ED \| 32 (56%) \| 13 (38%) \| 0.130 \| 82 (67%) \| 48 (55%) \| 0.113 \| \| Deterioration in ED \| 9 (16%) \| 3 (9%) \| 0.524 \| 18 (15%) \| 4 (5%) \| 0.022 \| \| **Received adrenaline** \|  \|  \|  \|  \|  \|  \| \| Any time \| 51 (89%) \| 11 (32%) \| <0.001 \| 109 (89%) \| 36 (41%) \| <0.001 \| \| Pre-hospital \| 31 (54%) \| 7 (21%) \| 0.002 \| 63 (51%) \| 13 (15%) \| <0.001 \| \| ED \| 5 (15%) \| 23 (40%) \| 0.011 \| 26 (30%) \| 57 (46%) \| 0.022 \| \| **Total adrenaline doses** \|  \|  \| <0.001 \|  \|  \| <0.001 \| \| 0 \| 6 (11%) \| 23 (68%) \|  \| 14 (11%) \| 51 (59%) \|  \| \| 1 \| 41 (72%) \| 8 (24%) \|  \| 73 (59%) \| 30 (34%) \|  \| \| ≥2 \| 10 (18%) \| 3 (6%) \|  \| 36 (30%) \| 6 (6%) \|  \| \| **Presented in business hours (Monday-Friday 8am- 4pm)** \| 24 (42%) \| 11 (32%) \| 0.382 \| 65 (53%) \| 26 (30%) \| 0.001 \| \| **Length of stay in ED/hospital** \|  \|  \| 0.047 \|  \|  \| 0.004 \| \| <4 hours \| 21 (37%) \| 22 (65%) \|  \| 28 (23%) \| 35 (40%) \|  \| \| 4-12 hours \| 16 (28%) \| 7 (21%) \|  \| 59 (48%) \| 42 (48%) \|  \| \| 12-24 hours \| 18 (32%) \| 4 (12%) \|  \| 28 (23%) \| 7 (8%) \|  \| \| 24+ hours \| 2 (4%) \| 1 (3%) \|  \| 8 (7%) \| 3 (3%) \|  \| \| **Discharge Unit** \|  \|  \| 0.021 \|  \|  \| 0.367 \| \| ED \| 33 (58%) \| 28 (82%) \|  \| 114 (93%) \| 84 (97%) \|  \| \| Inpatient Unit \| 24 (42%) \| 6 (18%) \|  \| 9 (7%) \| 3 (3%) \|  \| |
| --- | --- | --- | --- | --- | --- | --- | --- | --- | --- | --- | --- | --- | --- | --- | --- | --- | --- | --- | --- | --- | --- | --- | --- | --- | --- | --- | --- | --- | --- | --- | --- | --- | --- | --- | --- | --- | --- | --- | --- | --- | --- | --- | --- | --- | --- | --- | --- | --- | --- | --- | --- | --- | --- | --- | --- | --- | --- | --- | --- | --- | --- | --- | --- | --- | --- | --- | --- | --- | --- | --- | --- | --- | --- | --- | --- | --- | --- | --- | --- | --- | --- | --- | --- | --- | --- | --- | --- | --- | --- | --- | --- | --- | --- | --- | --- | --- | --- | --- | --- | --- | --- | --- | --- | --- | --- | --- | --- | --- | --- | --- | --- | --- | --- | --- | --- | --- | --- | --- | --- | --- | --- | --- | --- | --- | --- | --- | --- | --- | --- | --- | --- | --- | --- | --- | --- | --- | --- | --- | --- | --- | --- | --- | --- | --- | --- | --- | --- | --- | --- | --- | --- | --- | --- | --- | --- | --- | --- | --- | --- | --- | --- | --- | --- | --- | --- | --- | --- | --- | --- | --- | --- | --- | --- | --- | --- | --- | --- | --- | --- | --- | --- | --- | --- | --- | --- | --- | --- | --- | --- | --- | --- | --- | --- | --- | --- | --- | --- | --- | --- | --- | --- | --- | --- | --- | --- | --- | --- | --- | --- | --- | --- | --- | --- | --- | --- | --- | --- | --- | --- | --- | --- | --- | --- | --- | --- | --- | --- | --- | --- | --- | --- | --- | --- | --- | --- | --- | --- | --- | --- | --- | --- | --- | --- | --- | --- | --- | --- | --- | --- | --- | --- | --- | --- | --- | --- | --- | --- | --- | --- | --- | --- | --- | --- | --- | --- | --- | --- | --- | --- | --- | --- | --- | --- | --- | --- | --- | --- | --- | --- | --- | --- | --- | --- | --- | --- | --- | --- | --- | --- | --- | --- | --- | --- | --- |

† Cohort excludes those where provision of AAI or AAP on discharge was not indicated or required
