# Supplementary material for: A retrospective audit of adult and paediatric anaphylaxis management from two Australian metropolitan mixed emergency departments
Source: BMC Emerg Med. 2024 Apr 17;24:67. doi: 10.1186/s12873-024-00966-3 (PMC11022440; doi:10.1186/s12873-024-00966-3)
Supplement: Supplementary file 5 — Additional file 5. Supplemental Table 3. Factors Associated with supply of Allergy/Anaphylaxis Action Plans (AAP) on discharge from hospital in paediatric and adult patients. [file 12873_2024_966_MOESM5_ESM.docx]

**Supplemental Table 3. Factors Associated with supply of Allergy/Anaphylaxis Action Plans (AAP) on discharge from hospital in paediatric and adult patients**

| \|  \| **Paediatric** \| \| \| **Adult** \| \| \| \| --- \| --- \| --- \| --- \| --- \| --- \| --- \| \|  \| **AAP supplied†** \| \| **p-value** \| **AAP supplied†** \| \| **p-value** \| \|  \| Yes \| No \|  \| Yes \| No \|  \| \|  \| N= 30 \| N=60 \|  \| N=61 \| N=145 \|  \| \| **Sex** \|  \|  \| 1.000 \|  \|  \| 0.218 \| \| Male \| 15 (50%) \| 31 (52%) \|  \| 30 (49%) \| 57 (39%) \|  \| \| Female \| 15 (50%) \| 29 (48%) \|  \| 31 (51%) \| 88 (61%) \|  \| \| **History of Anaphylaxis** \| 12 (40%) \| 40 (67%) \| 0.023 \| 20 (33%) \| 81 (56%) \| 0.004 \| \| **Comorbid conditions** \|  \|  \|  \|  \|  \|  \| \| Asthma \| 10 (33%) \| 20 (33%) \| 1.000 \| 22 (36%) \| 38 (26%) \| 0.180 \| \| Eczema \| 6 (20%) \| 11 (18%) \| 1.000 \| 2 (3%) \| 8 (6%) \| 0.727 \| \| Allergic rhinitis \| 0 (0%) \| 3 (5%) \| 0.548 \| 3 (5%) \| 9 (6%) \| 1.000 \| \| Cardiovascular disease \| 1 (3%) \| 0 (0%) \| 0.333 \| 16 (26%) \| 24 (17%) \| 0.124 \| \| Cognitive impairment \| 1 (3%) \| 0 (0%) \| 0.333 \| 0 (0%) \| 3 (2%) \| 0.556 \| \| Substance abuse \|  \|  \| . \| 2 (3%) \| 8 (6%) \| 0.727 \| \| **Reacted to previously identified trigger** \| 10 (34%) \| 31 (62%) \| 0.022 \| 19 (40%) \| 51 (47%) \| 0.390 \| \| **Suspected Trigger** \|  \|  \| 0.165 \|  \|  \| 0.031 \| \| Food \| 24 (80%) \| 39 (65%) \|  \| 24 (39%) \| 64 (44%) \|  \| \| Venom \| 3 (10%) \| 3 (5%) \|  \| 20 (33%) \| 22 (15%) \|  \| \| Unknown \| 2 (7%) \| 14 (23%) \|  \| 15 (25%) \| 46 (32%) \|  \| \| Other \| 1 (3%) \| 4 (7%) \|  \| 2 (3%) \| 13 (9%) \|  \| \| **Anaphylaxis symptoms** \|  \|  \|  \|  \|  \|  \| \| Pre-hospital \| 25 (83%) \| 54 (90%) \| 0.496 \| 57 (93%) \| 132 (91%) \| 0.783 \| \| On arrival to ED \| 22 (73%) \| 23 (38%) \| 0.003 \| 45 (74%) \| 79 (54%) \| 0.012 \| \| Deterioration in ED \| 9 (30%) \| 4 (7%) \| 0.008 \| 13 (21%) \| 9 (6%) \| 0.003 \| \| **Received adrenaline** \|  \|  \|  \|  \|  \|  \| \| Any time \| 27 (90%) \| 33 (55%) \| 0.001 \| 56 (92%) \| 88 (61%) \| <0.001 \| \| Pre-hospital \| 10 (33%) \| 25 (42%) \| 0.498 \| 24 (39%) \| 49 (34%) \| 0.524 \| \| ED \| 18 (60%) \| 10 (17%) \| <0.001 \| 40 (66%) \| 44 (30%) \| <0.001 \| \| **Total adrenaline doses** \|  \|  \| 0.001 \|  \|  \| <0.001 \| \| 0 \| 3 (10%) \| 27 (45%) \|  \| 5 (8%) \| 57 (39%) \|  \| \| 1 \| 20 (67%) \| 28 (47%) \|  \| 35 (57%) \| 72 (50%) \|  \| \| >2 \| 7 (23%) \| 5 (8%) \|  \| 21 (35%) \| 16 (12%) \|  \| \| **Presented in business hours (Mon-Fri 8am-4pm)** \| 16 (53%) \| 18 (30%) \| 0.039 \| 48 (79%) \| 38 (26%) \| <0.001 \| \| **Length of stay in ED/hospital** \|  \|  \| <0.001 \|  \|  \| <0.001 \| \| <4 hours \| 3 (10%) \| 36 (60%) \|  \| 9 (15%) \| 57 (39%) \|  \| \| 4-12 hours \| 6 (20%) \| 15 (25%) \|  \| 23 (38%) \| 71 (49%) \|  \| \| 12-24 hours \| 20 (67%) \| 7 (12%) \|  \| 24 (39%) \| 11 (8%) \|  \| \| 24+ hours \| 1 (3%) \| 2 (3%) \|  \| 5 (8%) \| 6 (4%) \|  \| \| **Discharge Unit** \|  \|  \|  \|  \|  \|  \| \| ED \| 8 (27%) \| 49 (82%) \| <0.001 \| 56 (92%) \| 138 (95%) \| 0.344 \| \| Inpatient Unit \| 22 (73%) \| 11 (18%) \|  \| 5 (8%) \| 7 (5%) \|  \| |
| --- | --- | --- | --- | --- | --- | --- | --- | --- | --- | --- | --- | --- | --- | --- | --- | --- | --- | --- | --- | --- | --- | --- | --- | --- | --- | --- | --- | --- | --- | --- | --- | --- | --- | --- | --- | --- | --- | --- | --- | --- | --- | --- | --- | --- | --- | --- | --- | --- | --- | --- | --- | --- | --- | --- | --- | --- | --- | --- | --- | --- | --- | --- | --- | --- | --- | --- | --- | --- | --- | --- | --- | --- | --- | --- | --- | --- | --- | --- | --- | --- | --- | --- | --- | --- | --- | --- | --- | --- | --- | --- | --- | --- | --- | --- | --- | --- | --- | --- | --- | --- | --- | --- | --- | --- | --- | --- | --- | --- | --- | --- | --- | --- | --- | --- | --- | --- | --- | --- | --- | --- | --- | --- | --- | --- | --- | --- | --- | --- | --- | --- | --- | --- | --- | --- | --- | --- | --- | --- | --- | --- | --- | --- | --- | --- | --- | --- | --- | --- | --- | --- | --- | --- | --- | --- | --- | --- | --- | --- | --- | --- | --- | --- | --- | --- | --- | --- | --- | --- | --- | --- | --- | --- | --- | --- | --- | --- | --- | --- | --- | --- | --- | --- | --- | --- | --- | --- | --- | --- | --- | --- | --- | --- | --- | --- | --- | --- | --- | --- | --- | --- | --- | --- | --- | --- | --- | --- | --- | --- | --- | --- | --- | --- | --- | --- | --- | --- | --- | --- | --- | --- | --- | --- | --- | --- | --- | --- | --- | --- | --- | --- | --- | --- | --- | --- | --- | --- | --- | --- | --- | --- | --- | --- | --- | --- | --- | --- | --- | --- | --- | --- | --- | --- | --- | --- | --- | --- | --- | --- | --- | --- | --- | --- | --- | --- | --- | --- | --- | --- | --- | --- | --- | --- | --- | --- | --- | --- | --- | --- | --- | --- | --- | --- | --- | --- | --- | --- | --- | --- | --- | --- | --- | --- | --- | --- |

† Cohort excludes those where provision of AAI or AAP on discharge was not indicated or required
